# Editorial: Artificial intelligence in visual inspection

**DOI:** 10.3389/frai.2025.1715198

**Published:** 2025-10-31

**Authors:** Yanlong Cao, Zhijie Xu, Wenhan Zeng, Yuanping Xu

**Affiliations:** ^1^School of Mechanical Engineering, Zhejiang University, Hangzhou, China; ^2^State Key Laboratory of Fluid Power and Mechatronic Systems, Zhejiang University, Hangzhou, China; ^3^Xi'an Jiaotong-Liverpool University, Shuzhou, China; ^4^University of Huddersfield, Huddersfield, United Kingdom; ^5^Chengdu University of Information Technology, Chengdu, China

**Keywords:** AI-driven visual inspection, robustness, hybrid models, real-world applicability, explainable AI (XAI)

The integration of Artificial Intelligence (AI) into visual inspection represents a paradigm shift, moving beyond human limitations to achieve unprecedented levels of accuracy, efficiency, and scalability. From manufacturing floors and busy roadways to medical clinics and scientific research, AI-driven vision systems are transforming how we interpret and act upon visual data. This Research Topic, “*Artificial Intelligence in Visual Inspection*,” brings together cutting-edge research that addresses the core challenges of this transformation: robustness in adverse conditions, real-world applicability, and the critical need for precision and trust, particularly in high-stakes fields like medicine.

The contributing articles exemplify a field that is rapidly maturing. They move beyond mere proof-of-concept demonstrations to offer sophisticated solutions for complex, real-world scenarios. A common thread is the innovative fusion of techniques—combining image enhancement with detection, leveraging hybrid model architectures, and integrating classical computer vision with deep learning—to create systems that are greater than the sum of their parts.

A significant challenge in real-world visual inspection is operating under suboptimal conditions. The article by Liu et al. tackles this head-on, addressing the critical problem of *drone-view object detection in low-light environments*. Their proposed parallel joint encoding network is a notable innovation, co-optimizing image enhancement (using Zero-DCE++) and object detection (using a lightweight YOLOv5) within a single framework. This bidirectional approach, enhanced by specialized modules for feature modulation, significantly improves robustness against noise and insufficient illumination, as demonstrated on benchmark nighttime drone datasets. This work is vital for extending the operational window of drones in applications like nighttime surveillance, search and rescue, and traffic monitoring.

Shifting from the skies to the streets, the application of AI for public safety is explored by Deshpande et al.. His work on *automatic rider helmet violation detection in Indian smart cities* tackles a pressing real-world problem. The proposed two-step pipeline smartly leverages the strengths of different tools: the NVIDIA TAO toolkit with DetectNet for efficient rider and vehicle identification, and YOLOv8 for accurate helmet and license plate detection. By creating a custom dataset for a complex, real-world scenario and achieving high accuracy, this research provides a practical, deployable blueprint for automated traffic enforcement, with the potential to save lives.

The most demanding domain for visual inspection is often medicine, where precision can be a matter of life and death. Three articles in this Research Topic address this with remarkable sophistication. First, Koshy and Anbarasi introduce *HMA-Net for breast ultrasound image segmentation*. Their hybrid framework masterfully combines a ConvMixer-based encoder with a ConvNeXT-based decoder, augmented by multihead attention. This architecture is specifically designed to capture both local textures and global contextual dependencies in ultrasound images, a key challenge due to their noisy and complex nature. The model's exceptional performance on standard datasets, achieving a Dice coefficient of over 99%, underscores its potential as a powerful tool for aiding the early and accurate detection of breast cancer.

Similarly, Mochurad enhances medical image segmentation by integrating classical and deep learning techniques. Her *approach for chest X-ray segmentation* addresses the perennial challenge of low contrast and overlapping structures by preprocessing images with Sobel and Scharr edge detection filters. This simple yet highly effective strategy provides the subsequent U-Net model with enhanced boundary information, guiding it to achieve superior accuracy in segmenting the lungs, heart, and clavicles. This work demonstrates that hybrid methodologies can yield significant gains without necessarily increasing model complexity.

Finally, Farhan et al. tackle the complexity of *3D brain tumor segmentation from MRI* with a strategic ensemble approach. Their dual-modality method moves beyond using single MRI sequences, instead combining complementary modalities (e.g., T1ce and FLAIR) to exploit their synergistic information. Furthermore, the incorporation of Grad-CAM visualizations to create an *XAI-MRI* system is a crucial step forward. It not only achieves high segmentation accuracy but also provides explainable AI (XAI) heatmaps, building essential trust with clinicians by making the model's decision-making process transparent and actionable in a diagnostic context.

In conclusion, the research presented in this Research Topic vividly illustrates that the future of visual inspection is intelligent, hybrid, and trustworthy. These studies show that the next frontier is not just about building more accurate models, but about crafting robust systems that can function in the real world, seamlessly combine diverse techniques, and, especially in medicine, explain their reasoning. The collective findings here provide a robust foundation for the next generation of AI-powered inspection systems that will enhance safety, improve healthcare outcomes, and drive industrial innovation. The journey from theoretical algorithm to practical tool is well underway, and the work showcased in this Research Topic is leading the way.

